# Phytoplankton in headwater streams: spatiotemporal patterns and underlying mechanisms

**DOI:** 10.3389/fpls.2023.1276289

**Published:** 2023-10-24

**Authors:** Chenjun Zeng, Ran Xing, Bensheng Huang, Xiangju Cheng, Wenqing Shi, Shufeng Liu

**Affiliations:** ^1^ School of Civil Engineering and Transportation, South China University of Technology, Guangzhou, China; ^2^ Guangdong Research Institute of Water Resources and Hydropower, Guangzhou, China; ^3^ School of Environmental Science and Engineering, Nanjing University of Information Science and Technology, Nanjing, China

**Keywords:** headwater stream, phytoplankton, nutrient, hydrodynamic, aquatic ecosystem

## Abstract

Phytoplankton are key members of river ecosystems wherein they influence and regulate the health of the local environment. Headwater streams are subject to minimal human activity and serve as the sources of rivers, generally exhibiting minimal pollution and strong hydrodynamic forces. To date, the characteristics of phytoplankton communities in headwater streams have remained poorly understood. This study aims to address this knowledge gap by comparing phytoplankton communities in headwater streams with those in plain rivers. The results demonstrated that within similar watershed sizes, lower levels of spatiotemporal variability were observed with respect to phytoplankton community as compared to plain rivers. Lower nutrient levels and strong hydrodynamics contribute to phytoplankton growth limitation in these streams, thereby reducing the levels of spatiotemporal variation. However, these conditions additionally contribute to greater phytoplankton diversity and consequent succession towards *Cyanophyta*. Overall, these results provide new insights into the dynamics of headwater stream ecosystems and support efforts for their ecological conservation.

## 1 Introduction

Headwater streams are the first-order confluence units in the watershed, wherein they exist as primary rivers formed via the accumulation of runoff from the surrounding catchment area. These headwater streams play an important role in transporting and delivering water and nutrients from the land to the downstream rivers (Marx et al., 2017; Baattrup-Pedersen et al., 2018; Petrin et al., 2023). Phytoplankton are fundamental primary producers in aquatic ecosystems, serving as the bedrock of the food web and playing a pivotal role in nutrient cycling and energy flow (Qiu et al., 2013; Taipale et al., 2016; Amaneesh et al., 2023). As phytoplankton can promptly respond to signals indicating changes in the aquatic environment, their community structure can serve as a valuable indicator of ecological quality (Li et al., 2019; Chiellini et al., 2020). As such, there is a need for research focused on phytoplankton communities in headwater streams in an effort to preserve the associated aquatic ecosystems.

Many environmental factors, such as water temperature, nutrient availability, and hydrodynamics can impact the structure of phytoplankton community (Garzke et al., 2019; Xu et al., 2021). Water temperature can impact phytoplankton cell size and growth rate (Grimaud et al., 2017). Within a favorable water temperature range, rising water temperatures correspond to higher rates of phytoplankton growth (Baker and Geider, 2021). Changes in nutrient availability can significantly impact phytoplankton community structure, with growth rates being related to nutrient absorption rates under suitable water temperature and pH conditions (Schulhof et al., 2019; Villanova et al., 2021; Du et al., 2023). Hydrodynamic conditions also play a role in shaping phytoplankton community, as many phytoplankton are prone to thrive under stagnant water conditions (Li et al., 2013). Thus, there might be significant variability in phytoplankton community structure across different habitats. Headwater streams are primarily localized in mountainous regions where they exhibit strong hydrodynamic forces and are subject to relatively little human activity. The primary factors influencing the structure of headwater stream phytoplankton communities have not been firmly established, which hinders the development of evidence-based conservation strategies for these ecosystems.

In this study, we investigated phytoplankton communities in typical representative headwater streams and compared them with plain rivers. The main objectives were to study the phytoplankton community in headwater streams and identify key environmental factors influencing their patterns. The findings may provide significant implications for the ecological preservation of headwater streams.

## 2 Materials and methods

### 2.1 Study area

**Figure 1:**
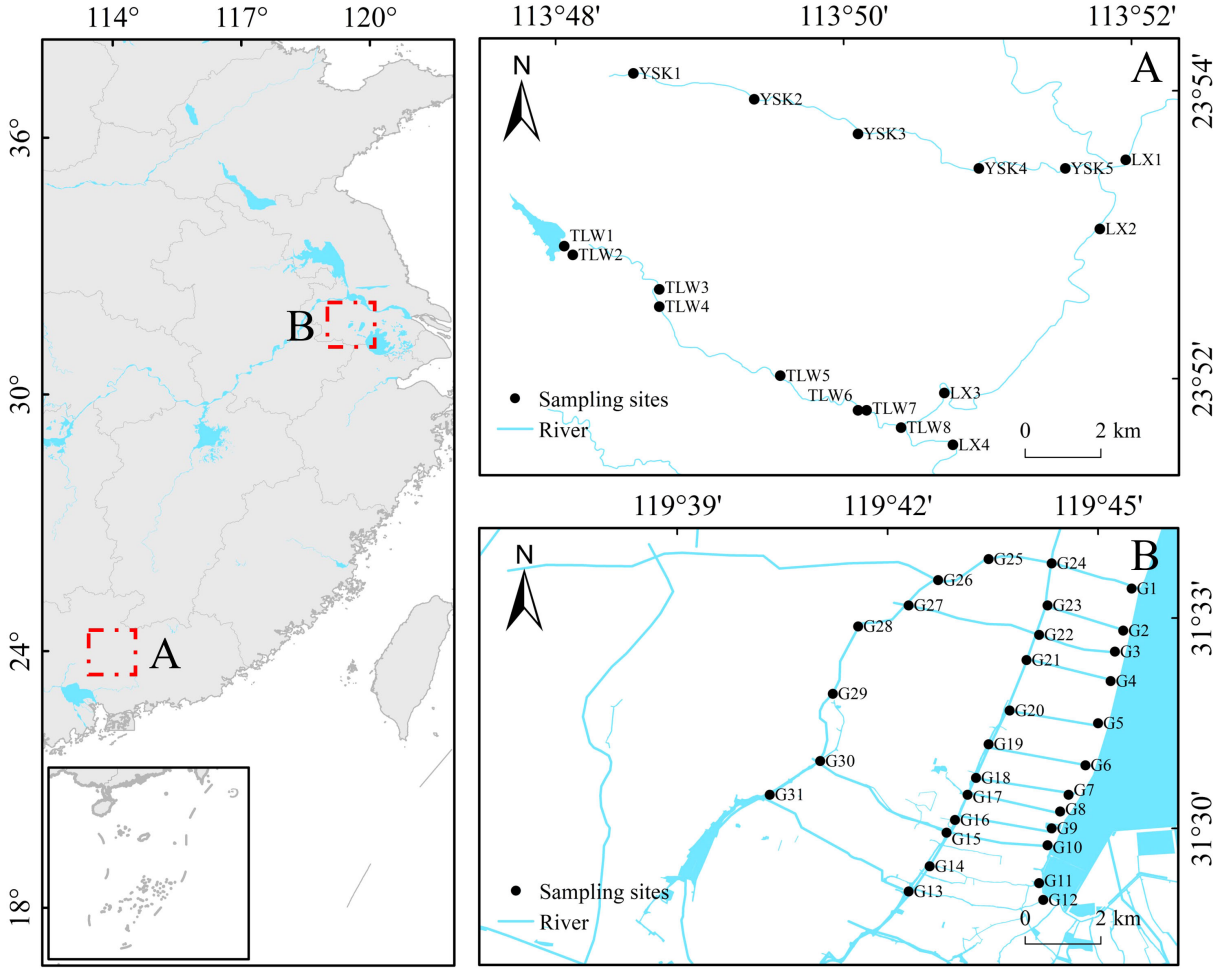
Location of study area and sampling sites. Headwater streams **(A)**; Plain rivers **(B)**.

This study was conducted in headwater streams (23°51’–23°54’ N, 113°48’–113°52’ E) of Pearl River, China (**Figure 1A**). The region experiences a subtropical maritime monsoon climate, characterized by long summers and warm winters, with annual temperatures ranging from 19°C to 24°C. The coldest temperatures are typically observed in January, averaging 16°C to 19°C, while the warmest temperatures occur in July, averaging 28°C to 29°C. Both headwater streams are situated in mountainous areas with an average slope of 2.6%. The study also included plain rivers (31°28’–31°34’ N, 119°40’–119°46’ E) in the watershed of Taihu Lake, China (**Figure 1B**), as the control. These rivers exhibited a flat terrain with an average slope of 0.04% and low flow velocity. The surrounding areas of these rivers were primarily characterized by residential zones and farmland. The region experiences a subtropical monsoon climate, with an average annual temperature of approximately 16°C. The Pearl River basin is predominantly mountainous, while the Taihu Lake basin is characterized by plains, making them commonly used subjects for studying headwater streams and plain rivers.

### 2.2 Field survey

Field sampling of headwater streams was conducted in June and December of 2022, including a total of 17 sampling sites (**Figure 1A**). Additionally, 31 sampling sites were established in the plain rivers (**Figure 1B**), and field sampling was conducted in January and July of 2022.

A multiparameter water quality meter (YSI ProQuatro, YSI Inc., USA) was used to measure water temperature, dissolved oxygen (DO), and pH at each site. Water samples were collected at 0.2 m below the surface using an organic glass water sampler with a capacity of 5 L. Subsequently, the water samples were transferred into 500 mL sample bottles for preservation and later water analyses.

Phytoplankton cell counting was performed on the collected surface water samples. For this, 1 L sample bottles to which 15 mL of Lugol’s iodine solution was added, aiding in the preservation of the samples for subsequent analysis.

### 2.3 Sample analyses

Total nitrogen (TN), total phosphorus (TP), nitrate, nitrite, ammonium, and soluble reactive phosphorus (SRP) levels were measured according to the Monitoring Analysis Method of Water and Wastewater. Water samples were passed through a 0.45 μm filter membrane (GF/F, Whatman) prior to the analyses of dissolved nutrients. Dissolved inorganic nitrogen (DIN) was calculated by summing up the concentrations of nitrate, nitrite, and ammonium values.

For phytoplankton analyses, the water sample with Lugol’s solution was left to settle for 24 hours. After sedimentation, the supernatant was siphoned off using a small-diameter silicone tube to obtain a final volume of 30 mL. The remaining water samples were thoroughly mixed, and 0.1 mL was taken with a pipette and placed on a counting chamber covered with a glass coverslip. The samples were then imaged at magnifications of 10 × and 40 × to identify the species and count the cells of each species. Counts for 20 randomly selected fields of view were obtained, with the average of three counts per field of view being determined and reported as phytoplankton cell density (cells/L).

The alpha diversity of phytoplankton community was measured using equations 1-3: (Chang et al., 2021):

Dominance index (*Y*) (McNaughton, 1967):


(1) $$Y=\frac{n_{i}}{N}f_{i}$$

where *n_i_* representing the number of individual phytoplankton in genus *i*; *N* represents the total number of individual phytoplankton at each point; *f_i_* represents the occurrence frequency of this species in the sample site.

Shannon-Wiener index (*H’*)


(2) $$H^{\prime }=-\sum_{i=1}^{S}\left(\frac{n_{i}}{N}\right)\ln(\frac{n_{i}}{N})$$

where *S* represents the number of phytoplankton genera per sampling point.

Pielou*’*s evenness index (*J*)


(3) $$J=\frac{H^{\prime }}{\ln S}$$

### 2.4 Statistical analysis

Spearman’s correlation analyses were used to analyze the correlations between environmental factors and phytoplankton abundance as well as alpha diversity values. Statistical analyses were performed using SPSS v22.0 (SPSS Inc., IL, USA). Results were compared using independent sample t-tests following the variance homogeneity tests. * *P*< 0.05, ** *P*< 0.01 and ***P< 0.001.

## 3 Results

### 3.1 Water temperature, DO, and pH levels

**Figure 2:**
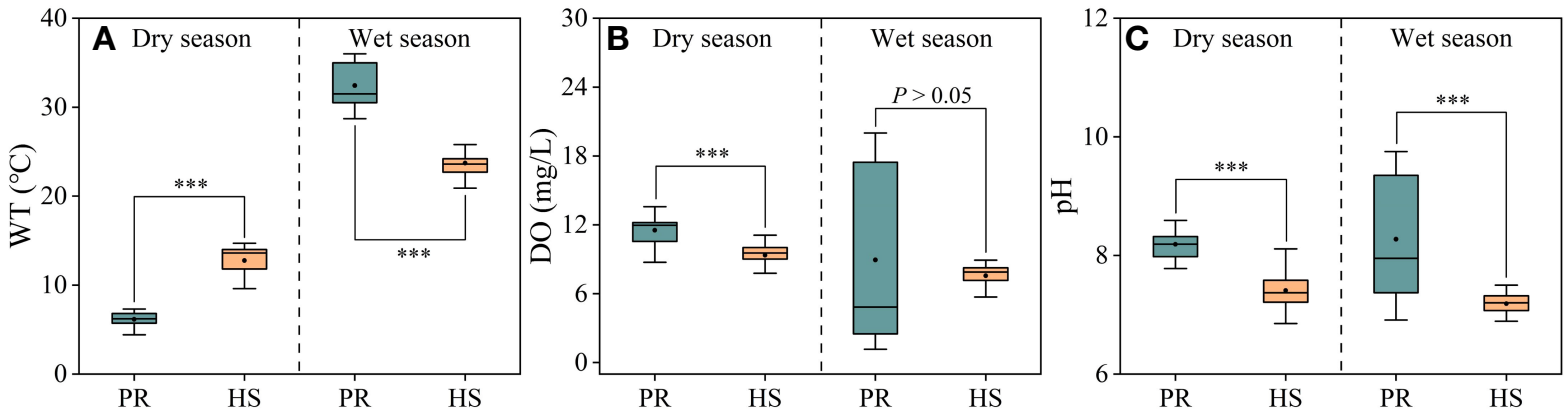
Characteristics of water temperature, DO and pH in the river flow. Water temperature **(A)**; DO **(B)**; pH **(C)**. PR, Plain rivers; HS, Headwater streams; WT, water temperature; DO, dissolved oxygen. ***P < 0.001.

Compared to the plain rivers, headwater streams showed relatively small fluctuations in water temperature, ranging from 9.6°C to 28.8°C. In the dry and wet seasons, the average water temperatures in headwater streams were 12.8°C and 23.7°C, respectively. On the other hand, the plain rivers experienced significant seasonal fluctuations, with average water temperatures of 6.2°C and 32.4°C during the dry and wet seasons, respectively (**Figure 2A**). There were no significant seasonal variations in average DO or pH values when comparing the headwater streams and the plain rivers. However, during the wet season, the plain rivers exhibited greater spatial fluctuations in DO (1.16–20.00 mg/L) and pH (6.9–9.8) (**Figures 2B, C**).

### 3.2 Water nutrients

**Figure 3:**
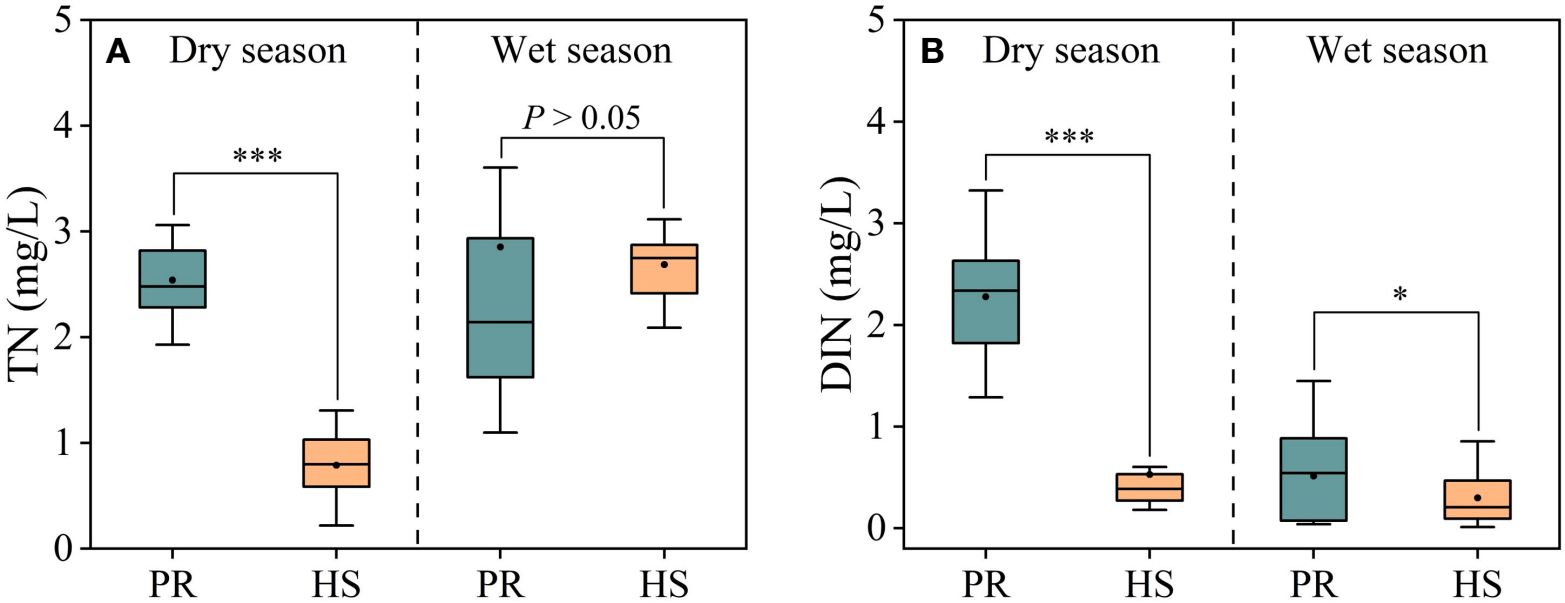
Characteristics of TN and DIN in the river flow. TN **(A)**; DIN **(B)**. *P < 0.05 and ***P < 0.001.

In headwater streams, significant seasonal variations in TN concentrations were observed, with average concentrations of 0.79 mg/L during the dry season and 2.69 mg/L during the wet season. In contrast, the plain rivers showed an average TN concentration of 2.54 mg/L during the dry season, while during the wet season, these concentrations fluctuated substantially, ranging from 1.10 to 3.60 mg/L, with an average of 2.85 mg/L (**Figure 3A**). On the other hand, no significant seasonal differences in DIN concentrations were observed in headwater streams, with an average of 0.53 mg/L during the dry season and 0.30 mg/L during the wet season, with respective ranges of 0.18–0.60 mg/L and 0.01–0.86 mg/L. In contrast, the plain rivers exhibited significant seasonal differences in DIN concentrations, with average values of 2.28 mg/L during the dry season and 0.51 mg/L during the wet season (**Figure 3B**).

**Figure 4:**
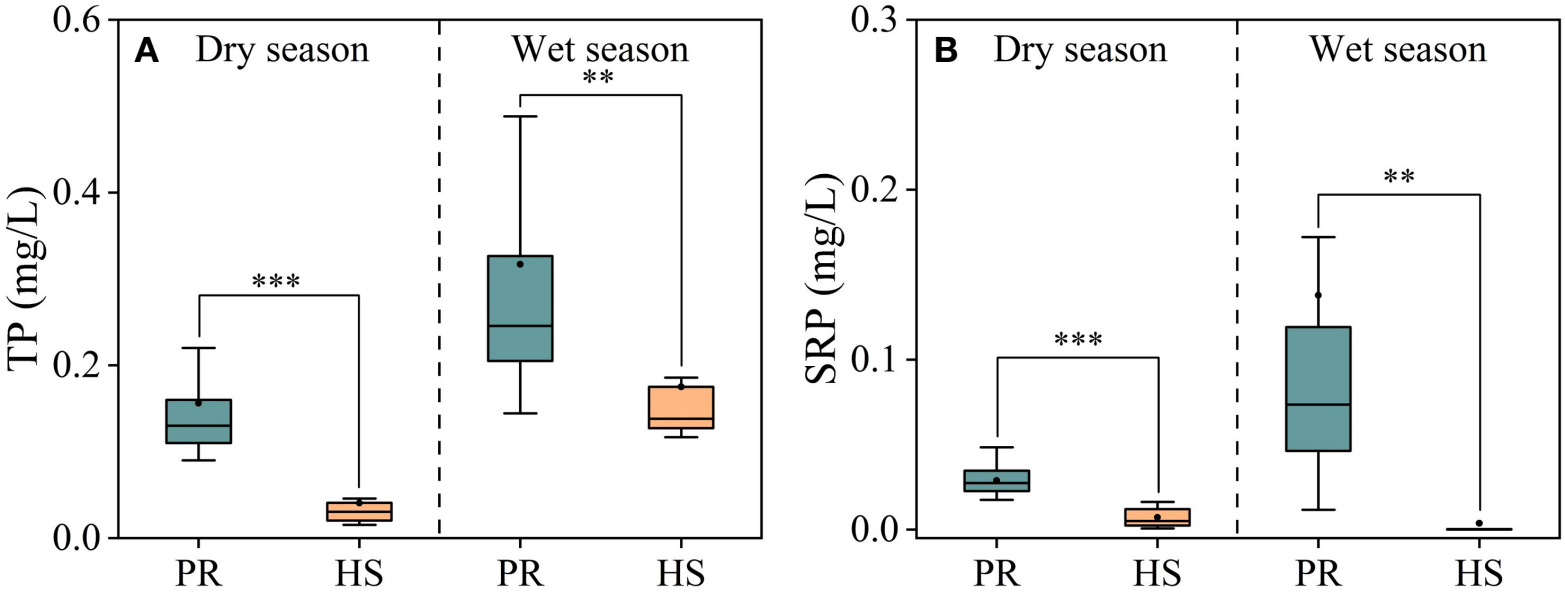
Characteristics of TP and SRP in the river flow. TP **(A)**; SRP **(B)**. **P < 0.01 and *** P < 0.001.

In general, headwater streams exhibited lower TP and SRP concentrations compared to the plain rivers, which aligns with the observed trend in TN concentrations. Notably, during the wet season, the plain rivers showed greater spatial fluctuations and higher overall TP and SRP concentrations (**Figures 4A, B**). Headwater streams displayed relatively low SRP concentrations, ranging from 0.001 to 0.016 mg/L during the dry season and mostly undetectable levels during the wet season. In contrast, the average SRP concentration in the plain rivers was 0.14 mg/L (0.01–0.17 mg/L) during the wet season, while the average TP concentration was 0.32 mg/L (0.14–0.52 mg/L).

### 3.3 Phytoplankton density and species

**Figure 5:**
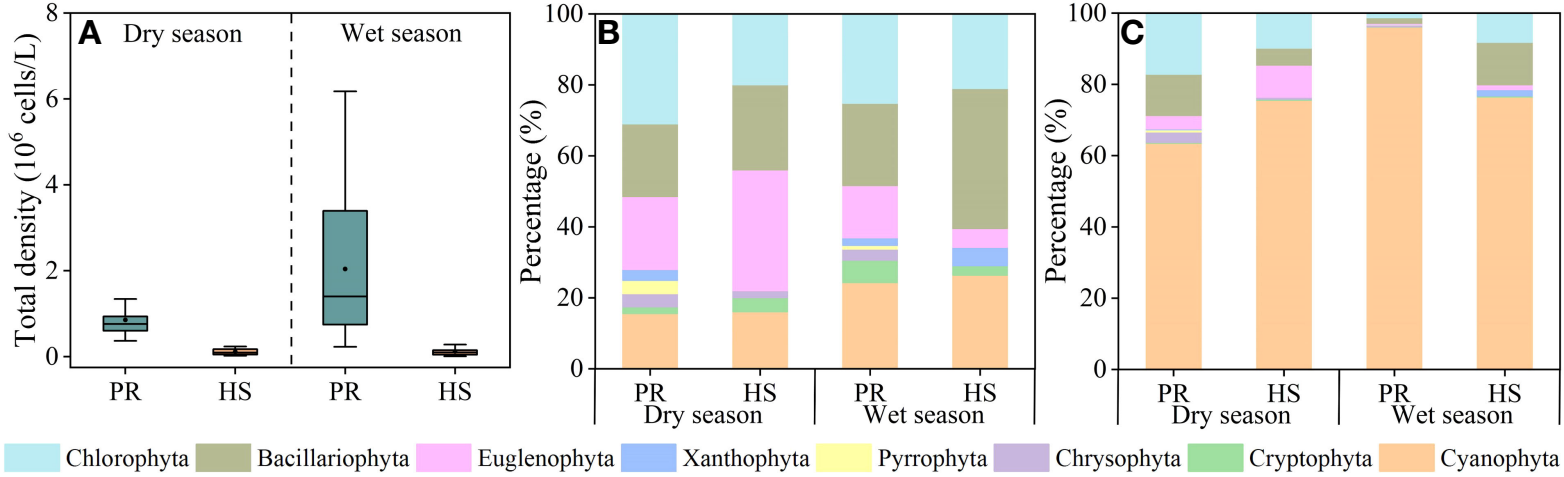
Characteristics of phytoplankton community in the river flow. phytoplankton density **(A)**; phytoplankton species **(B)**; phytoplankton density proportion **(C)**.

Headwater streams did not display any significant seasonal variability in phytoplankton density, maintaining an average of 1.18 × 10^5^ cells/L during the dry season (2.26 × 10^4^–2.38 × 10^5^ cells/L) and 1.25 × 10^5^ cells/L during the wet season (5.66 × 10^3^–5.32 × 10^5^ cells/L). In contrast, the plain rivers exhibited notable spatiotemporal differences in phytoplankton density. The average phytoplankton density in these rivers during the wet season was 2.04 × 10^6^ cells/L (2.28 × 10^5^–6.18 × 10^6^ cells/L), significantly higher than the average of 8.55 × 10^5^ cells/L during the dry season (3.68 × 10^5^–2.01 × 10^6^ cells/L) (**Figure 5A**).

In total, seven phytoplankton phyla were detected in the headwater streams, with an additional *Pyrrophyta* phylum being detected in samples collected from the plain rivers. The dominant phyla in both water systems included *Cyanophyta*, *Euglenophyta*, *Bacillariophyta*, and *Chlorophyta* (**Figure 5B**). In the headwater streams, 6 phyla, 30 genera, and 50 species of phytoplankton were detected during the dry season, while 6 phyla, 27 genera, and 38 species were detected during the wet season. In the plain rivers, 8 phyla, 54 genera, and 161 species were detected during the dry season, while 8 phyla, 42 genera, and 95 species were detected during the wet season (**Figure 5B**). During the dry season, the headwater streams were primarily dominated by *Cyanophyta*, *Chlorophyta*, and *Bacillariophyta*, accounting for 75.5%, 9.9%, and 9.0% of the overall phytoplankton community, respectively. Similarly, during the wet season, these dominant groups were *Cyanophyta*, *Bacillariophyta*, and *Chlorophyta*, with respective proportional abundance values of 76.4%, 11.9%, and 8.2%. In contrast, in the plain rivers, the dominant groups during the dry season were *Cyanophyta*, *Chlorophyta*, and *Bacillariophyta*, making up 63.4%, 17.2%, and 11.6% of the overall phytoplankton community. However, during the wet season, *Cyanophyta* dominated the plain rivers, representing a substantial 96.0% of the cell density (**Figure 5C**).

### 3.4 Phytoplankton diversity

**Figure 6:**
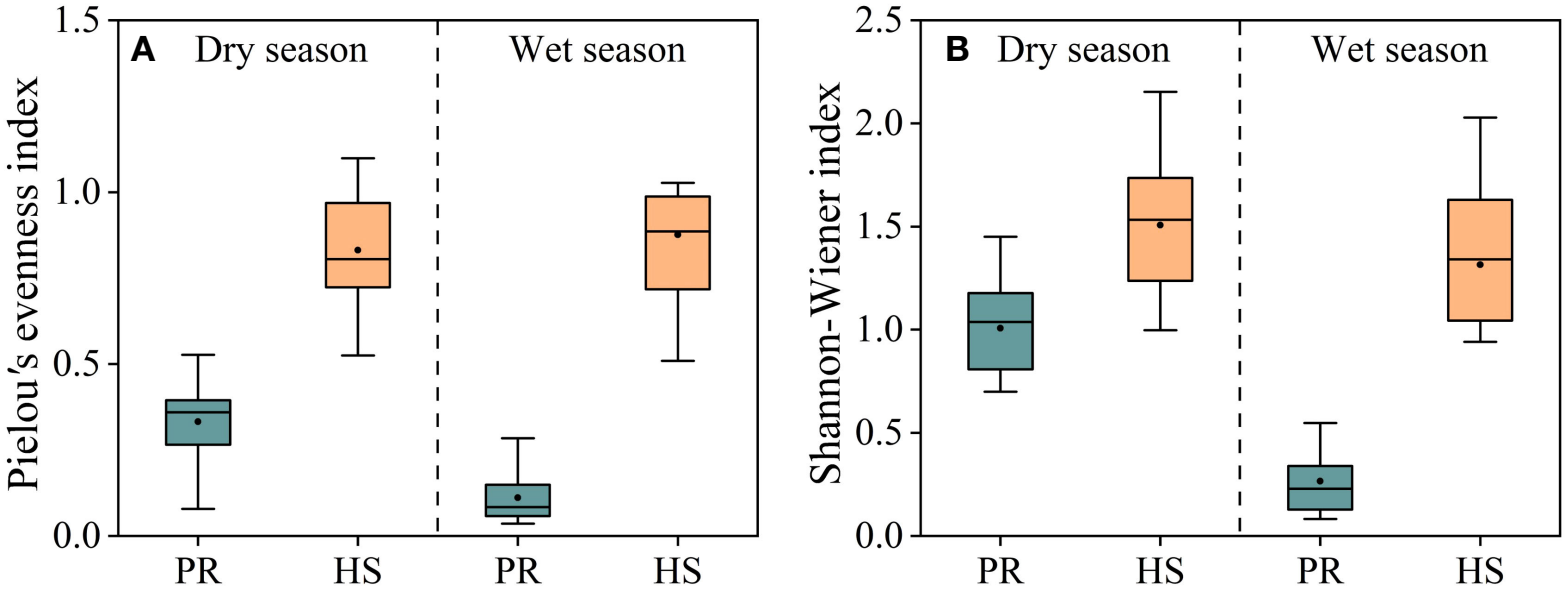
Pielou*’*s evenness and Shannon-Wiener indexes of phytoplankton in the river flow. Pielou*’*s evenness index **(A)**; Shannon-Wiener index **(B)**.

Both the Shannon-Wiener index and Pielou’s evenness index were significantly higher in headwater streams compared to those in the plain rivers, and there were no seasonal differences observed. In headwater streams, the average Pielou’s evenness index during the dry and wet seasons were 0.83 and 0.87, respectively, whereas the corresponding values for the plain rivers were 0.33 and 0.11. Similarly, the average Shannon-Wiener index values in headwater streams during the dry and wet seasons were 1.51 and 1.32, respectively, whereas the corresponding values for the plain rivers were 1.00 and 0.26 (**Figures 6A, B**).

## 4 Discussion

### 4.1 Headwater streams exhibit poor nutrient levels and strong hydrodynamics

Headwater streams generally exhibit lower levels of nutrients compared to the plain rivers, particularly in terms of SRP and DIN. The differences in nutrient concentrations in headwater streams are smaller during both the dry and wet seasons (**Figures 3**, **4**). This is primarily due to the limited human activities in headwater streams, resulting in lower external nutrient inputs. Additionally, the steeper slopes and stronger hydrodynamics in headwater streams impede nutrient accumulation (Lamb et al., 2017). In contrast, the plain rivers have weaker hydrodynamics and are affected by higher levels of nearby industrialization, agriculture, and urbanization. The uneven distribution of point and non-point pollution sources, such as agricultural runoff and domestic wastewater, also contributes to more significant spatial variability in nutrient levels within the plain rivers (Xiong et al., 2021; Tang et al., 2022). During the wet season, the TN levels in headwater streams increased significantly compared to the dry season, while there was no corresponding change in DIN levels. This can be attributed to frequent rainfalls during the wet season, which wash organic matter from the watershed into the streams (Panton et al., 2020; Croghan et al., 2021). Despite the increase in organic matter input, the elevated DO levels and less hydraulic residence time in these streams limit the conversion of organic nitrogen into inorganic nitrogen (Liu et al., 2021). As a result, there is no significant rise in inorganic nitrogen levels in these headwater streams (**Figure 3B**). The seasonal water temperature difference observed in headwater streams was 11.0°C, which was significantly lower than the 31.6°C in the plain rivers (**Figure 2A**). This discrepancy can be attributed to their geographic locations (Ding et al., 2016). There were relatively limited spatial differences in DO and pH among different sites, with the exception of the plain rivers during the wet season (**Figures 2B, C**).

### 4.2 Headwater streams display lower spatiotemporal variations in phytoplankton community

Diversity index were utilized in this study to assess the phytoplankton community structure (Wang et al., 2020; Hu et al., 2022). Both the Pielou’s evenness index and Shannon-Wiener index for headwater streams were significantly higher than those for the plain rivers (**Figure 6**), indicating a greater stability and even distribution of the phytoplankton community in headwater streams compared to the plain rivers. In headwater streams, there was relatively limited spatial variation in the phytoplankton community, with an average density during the wet season only 1.06-fold higher than that during the dry season. On the other hand, the plain rivers showed pronounced seasonal fluctuations in phytoplankton density, ranging from 3.68 × 10^5^ to 6.18 × 10^6^ cells/L, with an average density during the wet season that was 2.40-fold higher than that during the dry season. Additionally, significant spatial variability was evident during the wet season (**Figure 5A**). Overall, headwater streams displayed lower spatiotemporal variations in phytoplankton community composition compared to the plain rivers.

Within a specific range, there was a positive correlation observed between water temperature and phytoplankton growth rates (Grimaud et al., 2017; Gerhard et al., 2019). This correlation is particularly noticeable for *Cyanophyta*, as they exhibit greater water temperature sensitivity compared to other phytoplankton species (Elliott, 2010). Surprisingly, during the wet season, an 11.0°C increase in water temperature in headwater streams did not have any impact on phytoplankton density, and no apparent seasonal variations in the relative abundance of water temperature-sensitive *Cyanophyta* were observed in these headwater streams (**Figure 5C**). As a result, there were no significant correlations were found between water temperature, DO, pH, and phytoplankton density in headwater streams. These findings suggest that water temperature, DO, and pH might not be the primary influencing factors for the phytoplankton community in headwater streams.

During the wet season, both TP and TN levels in headwater streams increased compared to the dry season, while phytoplankton density remained relatively low (**Figure 5A**). This can be attributed to that SRP and DIN are the nutrient forms most readily utilized by aquatic organisms (Gomez-Velez et al., 2015; Chen et al., 2020). SRP and DIN concentrations in headwater streams remained consistently low without significant seasonal fluctuations (**Figures 3B**, **4B**). Hydrodynamics are also important factors influencing phytoplankton species composition. Larger *Bacillariophyta* cells are more likely to settle in the flow (Wang et al., 2018). In headwater streams with stronger hydrodynamic forces during the wet season, there were increased proportions of *Bacillariophyta* in the phytoplankton community (**Figures 5B, C**). However, the low nutrient availability limited phytoplankton growth in these headwater streams, resulting in no significant seasonal differences in phytoplankton density. Consequently, nutrient availability and hydrodynamic conditions were the primary factors influencing the phytoplankton community structure in headwater streams, leading to reduced seasonal variability in phytoplankton abundance.

During the dry season, there were no significant correlations between environmental factors and phytoplankton density in the plain rivers. However, during the wet season, phytoplankton density showed significant positive correlations with water temperature, DO, pH, TP, and SRP (**Supplementary Figure 1**). The phytoplankton community structure displayed pronounced seasonal differences, with *Cyanophyta* accounting for 63.4% of the phytoplankton density in the plain rivers during the dry season, but increasing to 96.0% during the wet season (**Figure 5C**). Previous studies have shown that *Cyanophyta* thrive in water temperatures between 25.0°C–35.0°C and exhibit heat-tolerant metabolic activity, making them able to tolerate water temperature fluctuations (Nalewajko and Murphy, 2001). During the wet season, the plain rivers experiences higher water temperatures conducive to *Cyanophyta* growth. These elevated water temperatures also enhance the nutrient acquisition rates by phytoplankton (Nalley et al., 2018), coupled with abundant nutrient supply in the plain rivers, facilitating *Cyanophyta* growth and their dominance during the wet season (**Supplementary Table 1**). Moreover, *Cyanophyta* have lower specific gravity, enabling them to cluster and proliferate under the lower flow velocity in the plain rivers (Huisman et al., 2018). As a result, plain rivers displayed lower spatiotemporal variations in phytoplankton community than headwater streams.

### 4.3 Implications for headwater stream conservation

Headwater streams play a crucial role as valuable natural resources, serving as vital water sources for energy production (Ferreira et al., 2019; Zhang et al., 2020). Although the water quality in the analyzed headwater streams was found to be in good condition, as evidenced by the Pielou’s evenness index for the phytoplankton communities (Ali and Khairy, 2016), However, it is essential to acknowledge that future resource development efforts could potentially impact the ecological health of these streams. At present, there is an increasing number of small hydropower stations being developed worldwide, particularly in headwater streams (Couto and Olden, 2018). The construction of small hydropower stations can reduce water flow velocity, leading to greater nutrient enrichment in the river (Alp et al., 2020; Lai et al., 2022). This disruption may have influences on phytoplankton communities in headwater streams. Moreover, while the impact of water temperature on phytoplankton communities in headwater streams is limited, the rising global temperatures may change this scenario (Hoegh-Guldberg et al., 2019). As nutrient concentration and water temperature increase, the risk of phytoplankton blooms in headwater streams could also increase. To ensure the health of these streams, it is essential to enhance monitoring and protection efforts while undertaking resource development. Strict regulations on river pollution and comprehensive assessments of the effects of resource development on headwater stream ecology are essential steps towards ensuring the sustainable health of these water bodies. In this study, headwater streams were situated in the Pearl River basin, while plain rivers were in the Taihu Lake basin. They were in separate watersheds, which may result in differences in phytoplankton communities due to their geographical locations. Moreover, their different field survey time may also influence the phytoplankton communities. Field surveys are still needed to collect more data in future.

## 5 Conclusions

In this study, we analyzed the characteristics of phytoplankton communities in headwater streams and evaluated their associations with environmental factors by comparing phytoplankton communities in headwater streams with those in plain rivers. The main findings are as follows:

(1) The phytoplankton communities in headwater streams exhibited greater diversity and lower spatiotemporal variability compared to those in plain rivers, with *Cyanophyta* being the dominant species.
(2) Low nutrient concentrations and strong hydrodynamics shape the structure of phytoplankton communities in headwater streams.
(3) These results offer valuable insights into the ecology feature of headwater streams, benefitting efforts to support their ecological conservation in the future.

## Data availability statement

The original contributions presented in the study are included in the article/**Supplementary Material**. Further inquiries can be directed to the corresponding author.

## Author contributions

CZ: Conceptualization, Methodology, Writing – original draft. RX: Methodology, Writing – review & editing. BH: Conceptualization, Writing – review & editing. XC: Writing – review & editing. WS: Writing – review & editing, Supervision. SL: Writing – review & editing.
