# Supplementary material for: Phytoplankton in headwater streams: spatiotemporal patterns and underlying mechanisms
Source: Front Plant Sci. 2023 Oct 24;14:1276289. doi: 10.3389/fpls.2023.1276289 (PMC10628446; doi:10.3389/fpls.2023.1276289)
Supplement: Supplementary file 1 [file DataSheet_1.docx]

Supplementary Material

# 1 Supplementary Figures and Tables

Supplementary Figure and Table legends

**Supplementary Table 1.** Dominant phytoplankton species during dry period and wet period

**Supplementary Figure 1.** Spearman’s correlation between environmental factors and the phytoplankton density as well as phytoplankton alpha diversity

**Supplementary Table 1.** Dominant phytoplankton species during dry period and wet period

| Category | Dominant species | Dominance index | | | |
| --- | --- | --- | --- | --- | --- |
|  |  | Plain River | | Headstream | |
|  |  | Dry season | Wet season | Dry season | Wet season |
| *Cyanophyta* | *Microcystis flos-aquae* | 0.231 | 0.117 | 0.501 | 0.514 |
|  | *Rhabdoderma lineare* | 0.079 | / | / | / |
|  | *Dactyloccopsis issegularis* | 0.026 | / | / | / |
|  | *Nostoc muscorum* | 0.028 | / | 0.043 | / |
|  | *Nostoc commune* | 0.024 | / | 0.046 | 0.047 |
|  | *Merismopedia trolleri* | 0.115 | / | / | / |
|  | *Chroococcus minor* | / | 0.792 | / | / |
| *Chlorophyta* | *Chlorella sp* | 0.036 | / | / | / |
|  | *Ulothrix zonata* | 0.054 | / | / | / |
|  | *Staurastrum crenulatum* | / | / | 0.05 | / |


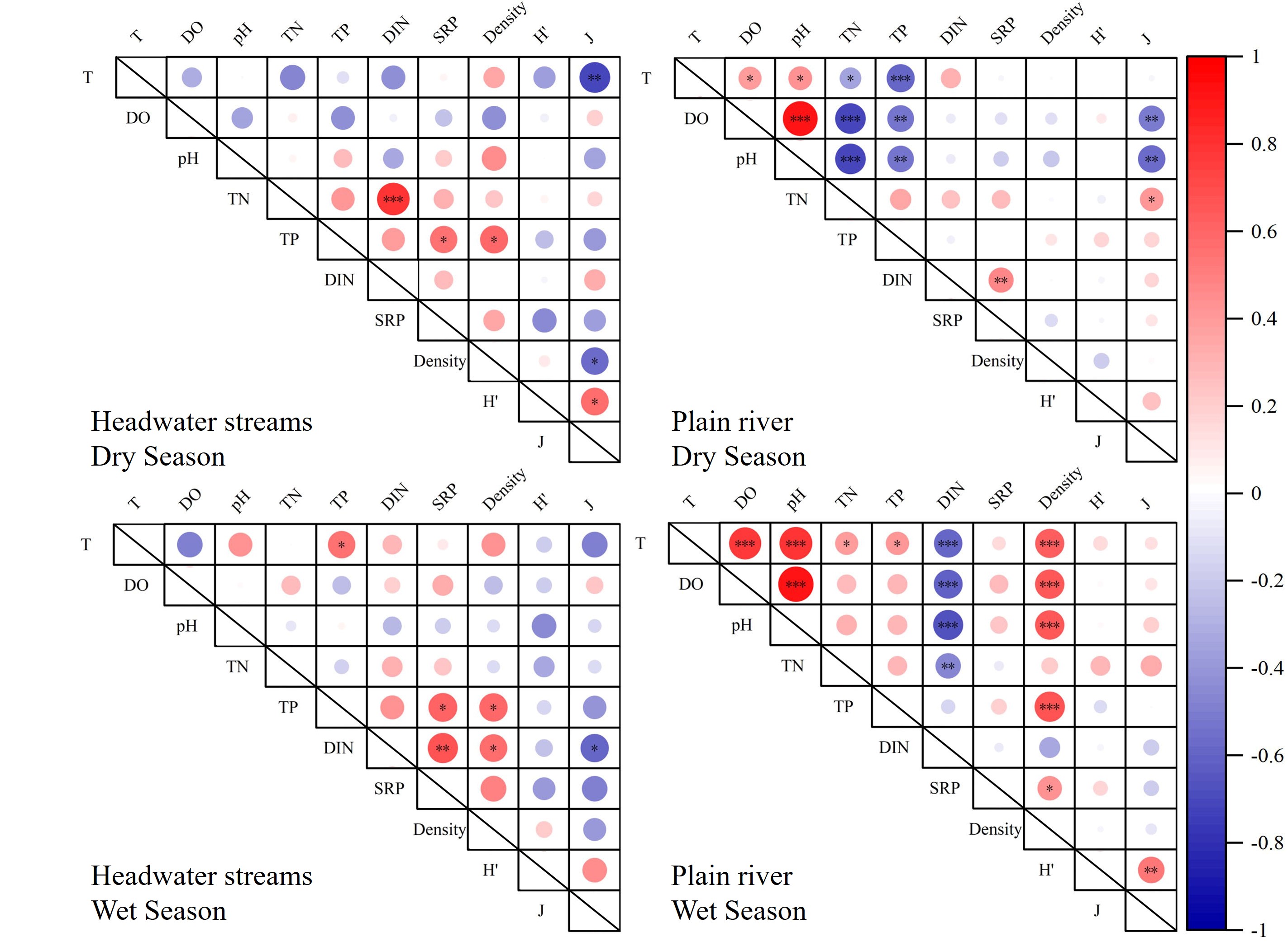


**Supplementary Figure 1.** Spearman’s correlation between environmental factors and the phytoplankton density as well as phytoplankton alpha diversity. * *P* < 0.05, ** *P* < 0.01 and ****P* < 0.001.
